# The Disruptive Effects of Pain on Complex Cognitive Performance and Executive Control

**DOI:** 10.1371/journal.pone.0083272

**Published:** 2013-12-30

**Authors:** Edmund Keogh, David J. Moore, Geoffrey B. Duggan, Stephen J. Payne, Christopher Eccleston

**Affiliations:** 1 Department of Psychology, University of Bath, Bath, United Kingdom; 2 Centre for Pain Research, University of Bath, Bath, United Kingdom; 3 Department of Computer Science, University of Bath, Bath, United Kingdom; University of Akron, United States of America

## Abstract

Pain interferes and disrupts attention. What is less clear is how pain affects performance on complex tasks, and the strategies used to ensure optimal outcomes. The aim of the current study was to examine the effect of pain on higher-order executive control processes involved in managing complex tasks. Sixty-two adult volunteers (40 female) completed two computer-based tasks: a breakfast making task and a word generation puzzle. Both were complex, involving executive control functions, including goal-directed planning and switching. Half of those recruited performed the tasks under conditions of thermal heat pain, and half with no accompanying pain. Whilst pain did not affect central performance on either task, it did have indirect effects. For the breakfast task, pain resulted in a decreased ability to multitask, with performance decrements found on the secondary task. However, no effects of pain were found on the processes thought to underpin this task. For the word generation puzzle, pain did not affect task performance, but did alter subjective accounts of the processes used to complete the task; pain affected the perceived allocation of time to the task, as well as switching perceptions. Sex differences were also found. When studying higher-order cognitive processes, pain-related interference effects are varied, and may result in subtle or indirect changes in cognition.

## Introduction

Pain serves as a warning of actual or potential harm [1]. To be useful pain needs to attract attention and override current concerns and goals, and investigations confirm that pain is very successful in achieving this [2], [3]. However, whilst initially adaptive, chronic interruption by pain leads to chronic distress and disability. Determining how pain achieves interruption, its consequences on behaviour, and the repair of attention after interruption are essential to a more comprehensive understanding of pain related interference.

Closer inspection of the influence of pain on attention reveals variable effects. Individual differences are likely to account for some of the variability in pain interference (e.g., age, sex, personality). In addition, features of the stimulus (e.g., novelty), as well as top-down factors (e.g., motivation, threat value), play a role [2]. Another consideration is that attention is a compound term for a range of processes [4], and the effects of pain might be selective [5]–[8]. For example, Moore et al. [6] found that experimentally induced thermal pain affected three (i.e., task switching, dual attention, attention span) out of seven types of attention. This suggests that tasks requiring higher-order processes, particularly executive control, are susceptible to pain interference [5], [9].

A strength with the approach adopted in many studies is that they distil attentional performance to very precise elements. However, on its own, this may also be a limitation because the tasks used (e.g., selectively attending to a rapid succession of letters) do not reflect the complex day-to-day activities we typically engage in. Indeed, even simple everyday tasks, such as preparing a meal, or shopping for food, actually involve a wide range of complex decisions. Unfortunately, we know little about the effects of pain on these more complex tasks of attention, and whether pain affects the strategies used to solve such problems. Whilst such issues have been considered in other health settings (e.g., neuropsychology, mental health [10], [11]), this has yet to translate to pain. This gap is surprising, particularly in light of more general concerns over the use of analgesics on everyday functioning (e.g., driving), which implies that pain has an effect on real world attention-based activities [12].

The primary aim of the current study was, therefore, to examine the effects of pain on complex tasks, which better reflect everyday attention and allow for consideration of the processes involved. To achieve this we chose two tasks, both of which have a multitasking component, and require planning and switching. Both tasks allow us to consider a number of different cognitive functions, and provide insight into the strategies used to complete them. It was expected that experimentally induced pain would have a detrimental effect on performance for both tasks, and result in changes in performance strategies that might be suboptimal. A second, exploratory, aim was to examine whether sex moderates pain interference effects. Male-female differences were considered because although there are known sex differences in pain, few studies have directly explored them in the context of pain-related attentional interference [13], [14].

## Methods

### Participants

Sixty two adult participants (40 female), with a mean age of 25 years (SD = 7.33), were recruited from the staff and student population of the University of Bath. Of these, 31 participants were randomly assigned to a pain condition (18 female) and 31 to a no pain control condition (22 female). All participants reported that they were not in pain upon arrival on the day of testing, had no existing chronic pain condition, were not taking analgesic medication, and had no skin complaints or skin sensitivity. All were paid a modest sum for participation.

### Pain manipulation

Participants in the non-pain condition completed the two tasks without any painful stimulation. Participants in the pain condition completed the tasks under pain stimulation, which was achieved using a Medoc PATHWAY – Advanced Thermal Stimulator (ATS). This equipment is designed for use in clinical and research settings, and induces pain through a metal plate, placed on the skin. The temperature is delivered and controlled through specialist hardware and software, designed for experimental purposes.

Individual pain thresholds were identified using a search protocol. The 30 mm×30 mm thermode was attached to the participant's right ankle. The thermode started from a baseline temperature of 32°C and participants altered the temperature using two buttons, one to increase the temperature and one to decrease the temperature. Participants were asked to increase the temperature to a level which was ‘just painful’. This was then monitored for 15 seconds and participants were asked again if this was ‘just painful’, if the participant reported that this level was still ‘just painful’ then this was taken to be the participant's threshold, if not then participants were asked to adjust the temperature to be ‘just painful’ and this check was performed again.

Once an individual thermal pain threshold was identified this was used to personalise a protocol for use during the experimental tasks. The temperature increased at a rate of 8°C/second to 1°C above each participant's set pain threshold (up to a maximum of 48°C; all participants with thresholds higher than this were tested with a temperature of 48°C). This was then oscillated between 1°C above and 1°C below the participant's pain threshold at 8°C/second for 10 oscillations before returning to the baseline temperature (32°C) at a rate of 8°C/second. This procedure was repeated on a continuous cycle throughout each task. This resulted in a series of painful episodes punctuated by a short period of non-pain. This procedure was used to ensure that participants did not habituate to the painful stimulus.

### Cognitive tasks - overview

Two tasks were included in the current study, both of which were conducted on a Dell Inspiron One 2205 touch screen computer, powered by a 3.00 GHz AMD Athlon II X2 250e processor running Windows 7. The tasks were chosen on the basis of results from previous research within our group, which suggest that measures of executive functioning, including aspects of working memory (in which multiple task demands are involved), may be particularly susceptible to pain. One task modelled breakfast making [15], and required planning and multitasking. The second task was a word generation puzzle [16], which also has competing elements, and has been used to examine how participants allocate time and resources across tasks.

### Breakfast task

The breakfast task used in the current study was the same as described by Craik and Bialystok [15]. It required participants to prepare a simulated breakfast, whilst concurrently setting places at a simulated table. Although the original Craik and Bialystok [15] study had three different levels of complexity, only the hardest of these conditions was used in the current study. This was because the task was originally designed for cognitive aging, and we wanted to avoid ceiling effects.

For the cooking component, participants were given five food items to prepare within a fixed time frame. Each item had a different cooking time (ranging between 2 and 5.5 minutes), and participants were required to calculate the optimum time to start each of the food items in order to ensure that they all reached their optimal cooked point simultaneously, without overcooking any item. Participants cooked each item by pressing a start and stop button. They were instructed to ensure that all items were ‘cooked’ when a visual timer, represented by a vertical bar beneath the food, reached zero. If the food was overcooked then the bar remained empty.

Whilst completing the breakfast component, participants were required to perform a secondary table setting task. A picture of a table was displayed with four empty places, onto which participants placed plates, knives, forks and spoons into their correct location. Once the table was fully set, the display was reset, and participants restarted the task.

Participants were informed that they should complete both tasks at the same time. They were told that the cooking task was their primary goal, but that they should also try and set as many table places as possible during the task. In terms of the display, participants could alternate between either cooking food items or the table setting task; in other words they could only see the food item or the table at any one point in time. Successful performance requires participants to maintain information in memory and switch between displays.

The core outcome variables associated with the (primary) cooking component of the breakfast task [2] were:

#### Discrepancy

This was based on the difference between actual cooking time taken and the ideal time taken for each food item. A low discrepancy score means that participants were better able to complete the cooking task on time. Because this task requires participants to switch from the table setting task and ensure the cooking task is completed on time, this variable is thought to have a strong prospective memory component.

#### Range of stop times

This was the difference in time between stopping the first and last items from cooking. A lower score indicates that all items were stopped around the same time. This outcome is used as a measure of global planning. It is also thought to involve aspects of working memory, and requires participants to remember the order in which different items are cooked, whilst keeping finishing times in mind.

### Average deviation in start times

This reflects participants' ability to start cooking each food item at the ideal time in order to ensure that all items finished cooking together. In order to maximise performance, participants need to calculate the ideal time delay before starting each food item based on its actual cooking time and how that in turn relates to the time required to cook the other items (i.e., the second item should be started 90 seconds after the first, the third item started 120 seconds after the first and 30 seconds after the second etc).

An average deviation in start times was calculated by taking the difference between the ideal starting time and actual starting time for food items 2–5. The first food item is not included in this calculation, as this is used as the starting reference point as it takes the longest time to cook. It is possible for these ideal start times to vary, however. For example, if the first item was started earlier that it should be, say 70 seconds rather than the optimal 90 seconds, then there is no longer a perfect start time for the third item. In this example, the third item still needs to be started after 120 seconds from the first item, but it should now be started 50 seconds (rather than 30) after the second item. Therefore the ideal start time for the third item can be expressed as the average start time between 120 after food item one, and 70+30 for food item two; in this example it would be 110 seconds after the first item. This principle can then be used to calculate the ideal start times for the fourth and fifth food items.

Once the deviation in start times was calculated for each item, an average deviation score was produced. A lower score is taken to reflect better performance (i.e., closer to ideal), and is thought to reflect a combination of planning (starting the relevant items at the correct time, best order) as well as prospective memory (remember the cooking times, estimating the best start time for each item).

In addition, there were also two outcomes associated with the table setting component of the task. These were:

#### Secondary task performance

This was calculated from the total number of places set at the table. It was used as a measure of task engagement and dual task performance.

#### Secondary task accuracy

An additional table setting outcome and calculated from the number of errors made when performing the table setting task (i.e., the number of times the participant tried to place an item in the wrong location). This can be used as a measure of participants overall accuracy at task performance.

### Overall processing speed

Finally, we had an overall performance outcome variable. This was called total task time, and was based on the total time participants took from starting the task to pressing the ‘all done’ button to end the task. A longer total task time (particularly associated with fewer total places set) can be used as a measure of global processing speed and dexterity.

### Word generation task

The second task used in the current study was a word generation task, used by Payne, Duggan and Neth [16]. This task required participants to generate words from two different sets of letters and was designed to assess participants' ability to allocate time across multiple tasks, and switching decisions in relation to task performance.

Participants were given two sets of letters, and asked to generate as many words as possible from each list. The two letter sequences were distinguished by the number of words that could be generated from them. The ‘Easy’ letter sequence was “L N A O I E T” which contained 53 words and the ‘Hard’ letter sequence was “E S I F L C E” which contained 23 words (see Payne et al. [16] for calculation of the potential word maximum for each letter sequence). Words had to be between two and seven letters in length, could not use the same letter twice, could not use letters from more than one letter sequence and were not allowed to be proper nouns or acronyms. Participants were told they could switch between the letter sequences as much or as little as they wanted but their aim should be to maximise the *total* number of words generated in the time provided.

The screen included a timer which counted down from 600 to 0, in 1 second intervals i.e., a total of 10 minutes. Two buttons, labelled “Sequence 1” and “Sequence 2”, were horizontally aligned at the top of the screen. Clicking on either Sequence button caused one of the letter sequences to be displayed across the middle of the screen. Half of the participants received the ‘Easy’ sequence when they clicked on Sequence 1 and the ‘Hard’ sequence when they clicked on Sequence 2. This was reversed for the remaining participants. Participants entered each word they generated by typing it into a textbox on the screen and then clicking Enter. As well as the word, the time taken to generate the word was also recorded.

There were three core outcome variables of interest that were derived from the word generation task. One related to overall task performance under the two difficulty conditions, whereas the second and third outcomes relate to the strategies used during the task itself; thus giving an insight as to what people are doing to maximise their moment by moment performance or “rate of return”. A final measure was administered after the task to gain self-estimates of performance.

The core outcome variables for this task were:

#### Word generation performance

This was the main outcome associated with task performance and calculated by summing the total number of correct words generated from each of the two word lists. This aspect of the task is very much associated with verbal fluency ability, and is thought to be a function of executive control in that it requires planning and monitoring elements to maximise the overall rate of return.

#### Time allocation

One method used to determine how people perform the task is to examine the amount of time they allocate to each version of the task. The optimal allocation of time would be whatever makes the average rate of return from each task equal (i.e., “matching”). For these particular groups of letters, Payne et al. [16] found that allocating approximately 75% of time to the easy task would be optimal in this sense, to maximise the overall return over 10 minutes. Therefore, the proportion of time spent on the easy task was calculated by dividing the time spent on the easy sequence of letters by the total task time. A higher score indicates that participants spent longer on the easy sequence condition compared to the difficult condition. Assuming that the optimal strategy of allocating 75% of time to the easy task is also the case for those in the current sample (both samples were recruited from UK Universities), the further the proportion of time is away from .75 is taken as indicative of a less optimal strategy.

#### Number of switches between tasks

This provided an indication of how participants allocated their time between tasks. The number of switches made between the easy and hard tasks was calculated and taken as a measure of switching behaviour. Although pain seems to produce greater switch costs [5], [6], the literature does not make a strong prediction about the effect of pain on number of switches. One hypothesis is that interruptions due to pain will provide more subtask boundaries. There is evidence that participants tend to switch at task boundaries [17], [18], and that task boundaries produced by sub-goal completion leads to task switching [16]. From this we would expect more switches between task types in the pain condition.

#### Subjective rating of performance

On completion of the task, participants also completed a short self-appraisal measure about their own task performance. They were asked to estimate how many words they thought they generated from each sequence, how long they thought they spent on each sequence (in time), how hard they found it to generate words from each sequence (using a range between 1 - very easy, and 7 - very hard) and how many times they thought they switched between letter sequences. The estimation scale was included because this task allows us to examine the strategies participants used on this task, and we were interested in knowing whether perceptions of the processes involved mapped onto actual behaviour.

Despite the fact that the amount of actual time allocated to the easy and difficult tasks are directly related (there was a set time allocated to the task), participants estimates of time allocated do not necessarily reflect the total available time. Therefore, to enable a comparison across participants, a proportion of estimated time allocated to the easy and difficult task was calculated using the following formula: [estimated time on easy task/(estimated time on easy task + estimated time on difficult task)].

### Verbal and non-verbal ability tasks

In order to ensure that there were no confounding differences between the pain and non-pain groups in either verbal or non-verbal ability, two additional measures were completed by all participants. The multiple choice set B of the Mill Hill Vocabulary Scale (MHVS) [19] was used as a measure of verbal ability, and Ravens Progressive Matrices (RPM) Advanced Set II was used as a non-verbal ability measure [20].

The MHVS consists of 36 multiple choice questions with a target word and 6 potential synonyms presented beneath, participants are instructed to simply underline the synonym which best relates to the target word. For the RPM there are 36 problems presented to participants, each consists of a target pattern at the top of the page in a book. In the bottom right hand corner of each pattern is a missing section, participants are presented with 8 potential solutions beneath each pattern and are asked to indicate which of the solutions best complete the pattern. For each problem, for both the MHVS and RPM, one point is awarded for a correct response. Both measures are well utilized, psychometrically sound, tests of verbal/non-verbal ability [19], [20].

### Ethics Statement

Full ethical committee approval was granted from the Departmental Ethics Committee, Department of Psychology, University of Bath (Reference number: 12-002). All participants were provided with full details about the study, and gave informed written consent. The pain induction protocols described here comply with guidance from the International Association for the Study of Pain.

### Data access requests

Data access requests should be directed to the Bath Centre for Pain Research (email: pain@bath.ac.uk).

### Procedure

All participants were first required to complete the MHVS and the RPM. Participants in the pain group had their pain threshold calculated. Participants were then required to complete the breakfast task and word generation tasks under either the pain condition or non-pain condition, depending on which group they were assigned to. The order of the experimental tasks was counterbalanced between participants.

## Results

### Verbal and non-verbal ability tasks

Means and Standard deviations for the MHVS and RPM for both the pain and non-pain groups are presented in Table 1. To examine for possible group differences these scales were entered into two 2 (pain condition: pain vs. control) × 2 (sex: male vs. female) between-groups ANOVA's. For the MHVS, no main effect of pain F(1,58) = 2.33, p = .13, or sex F(1,58) = .95, p = .33, and no interaction between the two F(1,58) = .03, p = .86, was found. For the RPM, there was a main effect of sex F(1,58) = 7.53, p<.01, with males exhibiting higher scores (mean = 26.09) than females (mean = 21.58). However, there was no significant main effect of pain condition, F(1,58) = 1.60, p = .21, and the interaction between pain and sex was also non-significant F(1,58) = 3.85, p = .06. Importantly, therefore, this indicates that any effects subsequently found to be associated with pain condition are not likely to be due to group differences in verbal and non-verbal ability.

**Table 1:** Means and standard deviations for verbal and non-verbal measures, and the breakfast task.

	No pain				Pain			
Measure	Male		Female		Male		Female	
MHVS	17.00	(4.27)	17.95	(3.84)	18.62	(4.57)	20.00	(5.12)
RPM	23.22	(6.69)	22.05	(5.47)	28.08	(3.71)	21.00	(6.25)
Discrepancy*	126.25	(151.48)	95.24	(120.89)	173.60	(219.48)	187.76	(224.07)
Range*	61.00	(41.21)	73.20	(75.23)	69.50	(73.24)	52.85	(29.59)
Average start time deviation*	57.34	(38.29)	111.01	(79.73)	113.87	(83.67)	106.01	(76.87)
Table places set	44.75	(10.25)	34.83	(12.06)	31.60	(10.41)	33.12	(9.15)
Table setting errors	19.63	(6.50)	13.06	(8.23)	17.40	(6.88)	13.71	(5.97)
Total time	358.50	(38.91)	369.12	(52.93)	341.70	(35.79)	371.24	(52.88)

Note: MHVS  =  Mill Hill Vocabulary Scale; RPM  =  Ravens Progressive Matricies;

* =  data was transformed in main analysis, but raw scores are presented here.

### Breakfast task

#### Data screening

Data screening was initially conducted on data from the breakfast task. An initial examination revealed that ten participants did not complete the task correctly e.g., they had started the food in the wrong order or had not prepared any food items. These participants were, therefore, removed from analyses, reducing the total number to 25 in the control condition (17 female) and 27 in the pain condition (17 female).

All remaining data were checked to ensure that they met assumptions. Data were standardized and examined for outliers (using cut off scores ±3.29 [21]). There was one outlying data point for the ‘discrepancy’ outcome, two for the range outcome, one for table setting errors and two for the total time for completion. As the number of outliers was small we brought them in line with the other scores by increasing/decreasing them to one unit above/below the next highest/lowest data point [22] (we compared this approach to an analysis where outliers were removed, with no overall difference in results found). For the range scores, there were an additional ten individuals who had missing data; this was because they failed to stop cooking the individual items at the end, and instead selected the ‘all done’ button. The final sample size for the range scores was 21 (15 female) for the control, and 21 (13 female) for the pain condition. Given this results in a fairly low number of males caution should be used when interpreting any sex difference effects for the range variable.

Frequency analysis indicated that some of the outcome variables were not normally distributed. Data for discrepancy and range were subjected to logarithmic transformations, and start times to a square root transformation, all of which corrected skew. While transformed scores were used in the analysis, raw scores are presented below and in the tables to aid clarity.

Means and standard deviations are presented in Table 1. Given that a number of variables were to be analysed for each task, we have taken a cautious approach throughout, and discounted marginal effects (e.g., those between .05 and .10). All effects are reported, however, including those that were non-significant to enable a full understanding of results found. Where significant interactions are found, simple effects analysis was conducted, with alpha set at .0125 to control for Type 1 errors.

#### Cooking task analysis

For all performance measures a series of 2 (pain condition: pain vs. control) × 2 (sex: male vs. female) between-groups ANOVAs were conducted. The first analysis was conducted on the (transformed) *discrepancy* score. This was the difference between the ‘correct’ cooking time and the actual cooking time, and was taken as a measure of prospective memory. No significant main effects were found for either pain condition F(1,48) = .82, p = .37, or sex F(1,48) = .01, p = .92, or for the interaction between the two F(1,48) = .48, p = .49.

The second analysis examined the (transformed) *range of stop times*; calculated as the difference in stop time between the first and last stopped items, and is taken as a measure of global planning. A lower score indicates that the participant was able to follow the instructions to ensure that all items should be finished at approximately the same time. No significant main effects were found for pain condition F(1,38) = .01, p = .97, sex F(1,36) = .01, p = .96, or for the interaction between the two F(1,38) = .01, p = .96.

The third outcome measure was the (transformed) *average deviation in start times*, and reflects both prospective memory and specific planning behaviour. Analysis revealed no significant main effects of either pain condition F(1,48) = 1.37, p = .25, or sex F(1,48) = 1.14, p = .29, and there was no significant interaction between the two F(1,48) = 1.86, p = .18.

#### Table setting analysis

In addition to performance related to the primary cooking task, task engagement on the secondary table setting task was conducted using two 2×2 between-groups ANOVAs. The first ANOVA was on the number of table places set during the task, which revealed a significant main effect of pain condition F(1,48) = 5.75, p<.05. Individuals in the pain condition set the table significantly fewer times (32.56) than those in the control condition (38.00). There was no significant main effect of sex F(1,48) = 1.84, p = .18, and no significant interaction between pain condition and sex F(1,48) = 3.41, p = .07.

A second analysis was conducted on secondary task errors (i.e., the number of times the participant tried to place an item in the wrong location). A significant main effect of sex was found F(1,48) = 6.21, p<.05, with males producing more errors (mean = 18.39) than females (mean = 13.38). There was no significant effect found for pain condition F(1,48) = .15, p = .70, and no significant interaction F(1,48) = .49, p = .49.

#### Overall time to complete task

A final analysis was conducted on overall processing speed, and based on the total time from starting and finishing the task. No significant main effects were found for either pain condition F(1,46) = .02, p = .90, or sex F(1,46) = .80, p = .38, or for the interaction between the two F(1,46) = .68, p = .41.

### Word generation task

#### Data screening

For the word generation task, a similar screening procedure was conducted. Of the original 62 participants, one female from the pain condition was excluded from all analyses as she reported not to have understood the task. Due to experimenter error, three participants from the control condition did not receive the post-task recall questions; their data were, however, included in analyses of actual task performance. Two outliers (with standardized scores ±3.29) were found for the recalled number of switches, and so were reduced to the next highest value (as above). Distributions were checked and within acceptable parameters, so no data were transformed.

Means and standard deviations for performance during the word generation task and for participants' recall of their task performance are given in Tables 2 and 3.

**Table 2:** Means and standard deviations (in parenthesis) for actual task performance on the word generation task.

	No pain				Pain			
Actual Performance	Male		Female		Male		Female	
Words generated – easy task	22.56	(11.40)	22.18	(8.48)	24.62	(10.87)	24.94	(7.36)
Words generated – hard task	9.67	(5.24)	8.73	(5.31)	12.08	(4.09)	9.88	(3.30)
Time spent on easy task	358.10	(58.75)	360.64	(50.69)	317.21	(43.57)	355.93	(44.84)
Time spent on hard task	241.90	(58.75)	239.36	(50.69)	282.79	(43.57)	244.07	(44.84)
Proportion time spent on easy task	.60	(.10)	.60	(.08)	.53	(.07)	.59	(.07)
Number of switches	7.67	(6.67)	7.23	(5.01)	8.77	(3.94)	11.12	(4.88)

**Table 3:** Means and standard deviations (in parenthesis) for recalled task performance on the word generation task.

	No pain				Pain			
Recalled performance	Male		Female		Male		Female	
Estimated words generated – easy task	17.22	(8.66)	19.37	(7.49)	20.62	(6.64)	18.18	(5.40)
Estimated words generated – hard task	13.67	(8.41)	11.29	(6.72)	12.85	(2.94)	11.06	(4.63)
Estimated time spent on easy task	366.67	(153.79)	342.16	(140.18)	277.69	(80.12)	337.06	(94.13)
Estimated time spent on hard task	245.56	(110.47)	174.95	(90.95)	282.31	(97.74)	255.88	(96.96)
Estimated proportion time spent on easy task	.60	(.17)	.66	(.12)	.51	(.12)	.57	(.15)
Estimated number of switches	5.72	(3.88)	5.21	(2.37)	7.04	(3.28)	7.82	(3.99)
Perceived difficulty – easy task	4.00	(1.80)	3.37	(1.07)	2.31	(.95)	3.35	(1.11)
Perceived difficulty – hard task	4.89	(1.62)	5.32	(1.16)	4.85	(1.07)	5.53	(.87)

#### Words generated

The number of words generated during the task was analysed using a 2 (pain condition: pain vs. control) × 2 (sex: male vs. female) × 2 (task difficulty: easy vs. hard) ANOVA. Pain condition and sex were between groups factors, whereas task difficulty was the within-groups factor. A significant main effect of task difficulty was found F(1,57) = 172.63, p<.001, with more words generated in the easy condition (mean = 23.52) than in the hard task condition (mean = 9.90). No other significant main or interaction effects were found [pain F(1,57) = 1.56, p = .22; sex F(1,57) = .23, p = .64; sex × pain F(1,57) = .01, p = .93; sex × task difficulty F(1,57) = .57, p = .46; pain × task difficulty F(1,57) = .09, p = .76; pain × sex × task difficulty F(1,57) = .23, p = .64].

Recall for the number of words generated during the task was analysed using a 2 (pain condition: pain vs. control) × 2 (sex: male/female) × 2 (task difficulty: easy vs. hard) ANOVA. Participants recalled generating more words in the easy task (mean = 18.97) than the hard task (mean = 11.94) F(1,54) = 114.21, p<.001. There was a significant three-way interaction between pain condition × sex × task difficulty F(1,54) = 4.35, p<.05. All other effects were non-significant [sex F(1,54) = .46, p = .50; pain F(1,54) = .03, p = .86; sex × pain F(1,54) = .37, p = .55; sex by task difficulty F(1,54) = 2.43, p = .13; Pain × task difficulty F(1,54) = 1.72, p = .20].

**Figure 1:**
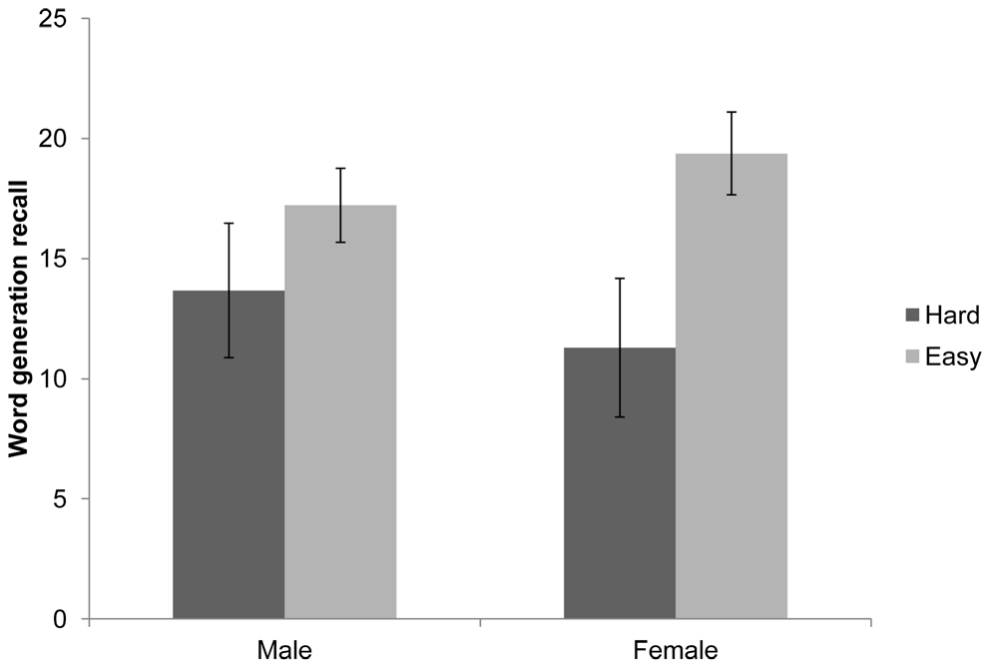
Effect of task difficulty and sex on memory for number of words generated amongst those in the no pain condition. (error bars reflect standard error of mean).

**Figure 2:**
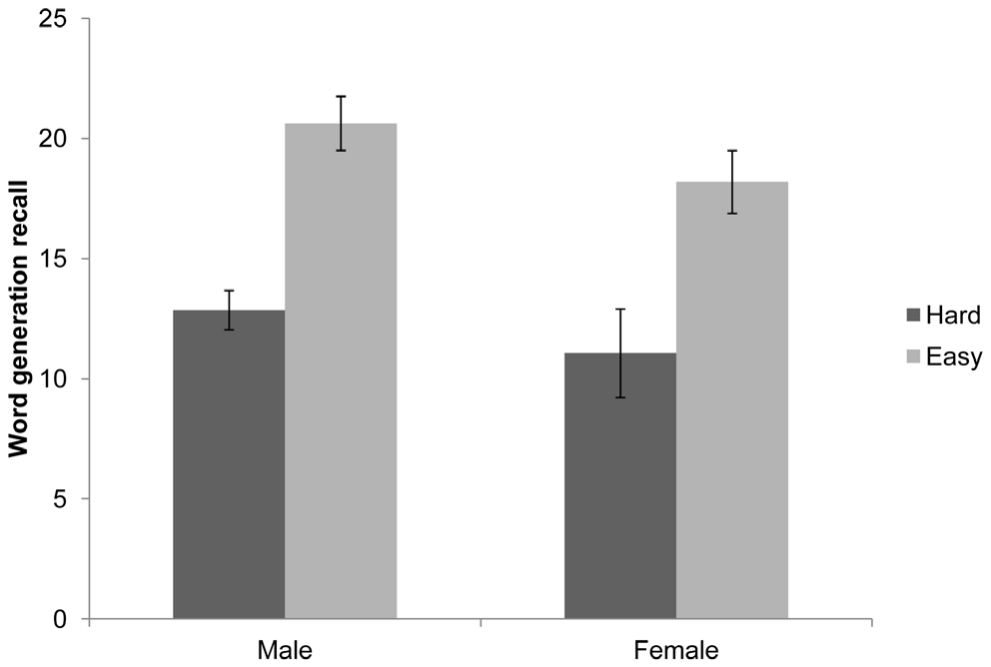
Effect of task difficulty and sex on memory for number of words generated amongst those in the pain condition. (error bars reflect standard error of mean).

In order to understand the nature of this 3-way interaction, separate ANOVAs were conducted for the control and pain conditions (see Figures 1 and 2). This indicated a significant sex × task difficulty interaction within the control condition F(1,26) = 5.09, p<.05, but not the pain condition F(1,28) = .19, p = .67. Follow-up simple effects analysis was therefore conducted amongst those allocated to the control condition, with alpha set at .0125. Females perceived the hard task to be significantly more difficult than the easy task F(1,26) = 50.51, p<.001, whereas males did not F(1,26) = 4.63, p>.0125. No significant differences between males and females were found for recall in either the easy or difficult task (p>.0125).

#### Time allocation

The proportion of time spent on the easy task was analysed using a 2 (pain condition: pain vs. control) × 2 (sex: male vs. female) between groups ANOVA. The effect of sex F(1,57) = 2.43, p = .12, pain condition F(1,57) = 2.97, p = .09, and the pain condition × sex interaction F(1,57) = .87, p = .18, were not significant.

Recall for the time spent on the easy task was analysed using a 2 (pain condition: pain vs. control) × 2 (sex: male vs. female) between-groups ANOVA. For this variable, the proportion of estimated time spent on the easy task was calculated. Participants in the pain condition recalled spending proportionally less time (mean = .54) on the easy task than those in the control condition (mean = .64) F(1,54) = 5.91, p<.05. The effect of sex F(1,54) = 2.41, p = .13, and the pain condition × sex interaction F(1,54) = .01, p = .93, were not significant.

#### Switching behaviour

The number of switches made between the two tasks was analysed using a 2 (pain condition: pain vs. control) × 2 (sex: male vs. female) between-groups ANOVA. There were non-significant effects of pain condition F(1,57) = 3.35, p = .07, and sex F(1,57) = .49, p = .49. The pain condition × sex interaction was also non-significant F(1,57) = 1.04, p = .31.

Recall for the number of switches between tasks was analysed using a 2 (pain condition: pain vs. control) × 2 (sex: male vs. female) between-groups ANOVA. A significant effect was found for pain condition F(1,54) = 4.60, p<.05, with participants in the pain condition recalling more task switches (mean = 7.48) than those in the control condition (mean = 5.38). There was no significant effect of sex F(1,54) = .02, p = .88, or significant interaction between pain condition × sex F(1,54) = .50, p = .48].

#### Perceived task difficulty

Post-task estimate of task difficulty was analysed using a 2 (pain condition: pain vs. control) × 2 (sex: male vs. female) × 2 (task difficulty: easy vs. hard). Perceived task difficulty was the within-groups variable, and pain condition and sex served as between-groups variables. A significant main effect of task difficulty was found F(1,54) = 105.41, p<.001; participants estimated that the easy task was easier (mean = 3.22) than the hard task (mean = 5.21). There was also a significant interaction between pain condition and task difficulty F(1,54) = 6.52, p<.05. The other effects were non-significant [pain F(1,54) = 2.15, p = .15; sex F(1,54) = 2.11, p = .15; sex × pain F(1,54) = 3.40, p = .07; sex × task difficulty F(1,54) = .90, p = .35; pain × sex × task difficulty F(1,54) = 3.73, p = .06].

**Figure 3:**
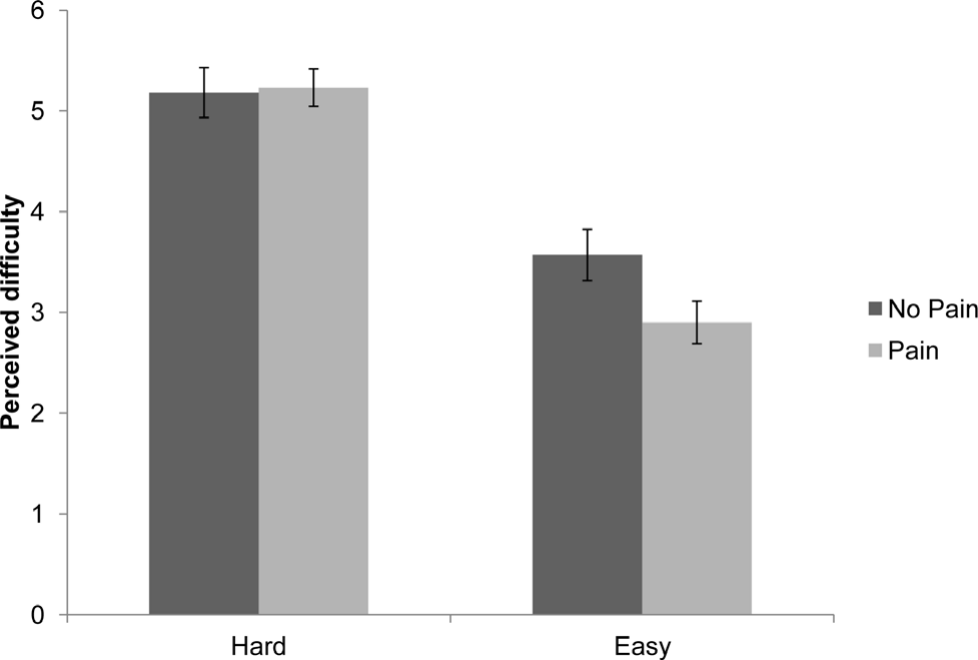
Effect of pain and task type on perceived difficulty levels during the word generation task. (error bars reflect standard error of mean).

In order to follow up the significant 2-way interaction, simple effects analyses were conducted (see Figure 3). This revealed that, as expected, the easy task was perceived to be significantly easier than the hard task by both the pain F(1,54) = 90.67, p<.001, and control groups F(1,54) = 27.20, p<.001. In addition, those in the pain condition rated the easy task as easier than those in the control condition F(1,54) = 6.82, p<.0125. There were no differences between the two groups for the hard task F(1,54) = 0.74, p = .79.

## Discussion

This study considered the interference effects of induced thermal pain on two complex tasks of attention. Both tasks involved higher-order executive functions to maximise performance, and enabled an investigation of the process involved in optimising performance. Whilst pain affected both tasks, the pattern of effects found was subtle and intriguing.

The breakfast task has planning and prospective memory aspects to it, and requires participants to multitask, switching between primary and secondary elements. Whilst pain was found to disrupt performance, this was not found for the primary cooking task, but instead affected performance on the secondary table setting task. Although it might be surprising to discover that performance on the central task remained intact during pain, this is consistent with previous findings. Using a basic dual task paradigm, we reported that laboratory-induced pain had a detrimental effect on a spatially peripheral task, whereas it actually improved performance on a central task [6].

One reason why primary task performance was spared during pain might be because attention is prioritized to this task. Anxiety is known to focus attention towards salient objects, resulting in reduced peripheral awareness [23], [24]. It is possible that pain has a similar effect on selection, and that when having to multitask, attention can be preferentially allocated to salient or motivationally important tasks, at the expense of peripheral or secondary tasks. Motivational focus is increasingly considered important in pain [2], [25], and if task goals are important it would be interesting to see whether a similar pattern would be found if the table setting component was made the primary task i.e., would we see a decline in planning and prospective memory processes in a ‘secondary’ cooking task?

The second core task used in the current study was the word generation puzzle. This task is closely related to verbal fluency ability, and thought to be under executive control due to its reliance on planning and monitoring [16]. Like the breakfast task, we did not find an overall effect of pain on task performance. However, when we examined the strategies used to conduct the task, pain-related differences were found in how participants viewed their own performance. Those in the pain condition recalled using a performance profile that reflected a poorer set of processing strategies i.e., poorer time allocation to the tasks, and a greater number of switches between tasks. This was despite there being no significant effect of pain on the actual processing strategies used. This indicates that pain may detrimentally alter a person's perceptions about the strategies they use to perform cognitively demanding tasks. Such beliefs might in turn elicit the use of alternative anticipatory top-down strategies to compensate for attentional interference. Such a view would be generally consistent with studies that show that working memory can help protect (or shield) people from the disruptive effects of pain [7], [26]. Interestingly, although actual process indicators for time allocation and switching behaviour failed to show group differences they approach significance and were in the same direction to self-reports. Whilst it is tempting to suggest that pain may have influenced the cognitive strategies used, the lack of statistical significance means such a interpretation is not possible here.

In addition to considering the effect of pain on performance, the current study also examined for sex differences in pain interference. Within the breakfast task, males made more errors on the secondary task, whereas in the word generation task pain seemed to remove a belief held by males that they performed equally well on both version of the task, despite there being no evidence for actual performance differences. Unfortunately, few studies directly consider sex differences in attentional interference, and so direct comparisons are not possible [14]. There are, however, well described sex differences in both pain and pain-related cognitions (e.g., pain catastrophizing; gender-role attributions [27], [28]), and there are similarities between the current findings and those in cognate areas. The breakfast task findings are broadly consistent with the popular (stereotypical) view that males are less able to multitask [29], although even here evidence is somewhat mixed [29]–[31]. The word generation results are more novel, but suggest pain may affect how males perceive their performance on cognitive tasks. It would be interesting to examine whether such differences translate to other tasks, as well as what the consequences of such belief might be on subsequent task performance.

Before considering further implications it is worth noting some limitations with the current study. One concern is the number of participants who were removed from the breakfast task prior to the main analysis. The main reasons were that participants failed to fully understand the task, or did not correctly perform task in the correct order. Although this was a task used in previous studies, it suggests that there may be issues with the task process that need to be addressed. A second issue relates to the decision to adopt a between-groups approach to the pain induction protocol, which bring issues associated with potential inter-group differences. The rationale for this choice was based on concerns surrounding whether participants would benefit from memory strategies in the word generation task. Whilst we believe that the results found here are novel, innovative, and warrant further interest, there is clearly a need for replication, and confirm the reliability of effects found. Future research may wish to consider utilizing alternative tasks, and utilizing within-groups designs to avoid some of the issues raised here.

Despite these potential limitations, there are also interesting implications to consider. For the first time we have successfully shown that pain interference effects can be investigated using complex tasks of cognition, that are not only conceptually closer to the type of experiences we have on a day-to-day basis (e.g., multitasking), but also allow us to consider the processes used to optimise performance. Whilst the benefits of experimental protocols are to control carefully the environment, as well as to enable us to determine causal effects through careful manipulation of core parameters, there is a need to ensure that there is translation to real world experiences. We have argued elsewhere [32] that it is important for laboratory-based pain induction studies to be supplemented with alternative methods for investigating everyday pain experiences. Here we suggest that it is equally important to extend the need for real world relevance to pain interference effects. There are well developed methods for investigating executive control in areas such as developmental psychopathology and neuropsychology that we may learn from [33], [34]. In addition, we may need to consider developing new tasks of everyday attentional interference, or perhaps even utilize technological developments in virtual reality and gaming that allow for simulation of real world events [35], [36]. By designing studies that combine these different approaches, we should not only be able to determine the specific facets of attention that are affected by pain, but also predict the type of everyday activities that are most likely to be affected by pain.

A second implication is that simple outcome measures of performance may not fully capture the way that pain affects people. By taking simple speed or accuracy outcomes as indicators of attentional performance, this may result in conclusions that some processes are less affected by pain. Indeed, one interpretation of the current findings is that pain has no effect on performance; the pain manipulation did not produce significant differences on either of the primary outcomes from the two tasks. However, closer examination reveals that wider contextual factors need to be considered. The breakfast task suggests that when in pain primary performance is maintained by directing attention away from secondary, less important goals. The word generation task suggests that the processes used may be modified or adapted to successfully complete a primary goal. Motivational factors, different strategies, as well as individual difference variables may also play a role in pain interference.

Finally, greater consideration of individual differences and in particular the potential for sex to moderate pain-related interference effects is required. These results they are amongst the first to directly examine for sex difference in attention and/or memory processes, and confirm that considering male-female variation in more detail may help understand why there are differences in the experience of pain.

In conclusion, this study is the first to consider interference effects of pain using more complex tasks of executive function. We have shown that the wider context is important, and that reliance on simple outcomes measures may be limited. Further consideration of strategies people use to perform these tasks when in pain, as well as the potential for individual differences, is required.
